# Tissue plasminogen activator mediates deleterious complement cascade activation in stroke

**DOI:** 10.1371/journal.pone.0180822

**Published:** 2017-07-10

**Authors:** Xue-Jun Zhao, Timothy M. Larkin, Molly A. Lauver, Saif Ahmad, Andrew F. Ducruet

**Affiliations:** 1 Department of Neurological Surgery, University of Pittsburgh, Pittsburgh, PA, United States of America; 2 Department of Neurosurgery, Barrow Neurological Institute, Phoenix, AZ, United States of America; Indian Institute of Integrative Medicine CSIR, INDIA

## Abstract

The use of intravenous tissue plasminogen activator (tPA) in the treatment of ischemic stroke is limited by its propensity to exacerbate brain edema and hemorrhage. The mechanisms underlying these deleterious effects of tPA remain incompletely understood. The purpose of this study was to delineate a pathway of tPA-mediated complement cascade activation in stroke and to determine whether complement inhibition ameliorates the adverse effects of post-ischemic tPA administration. We found that tPA promotes C3 cleavage both *in vitro* and in ischemic brain through a plasmin-mediated extrinsic pathway. Using cell culture models, we then showed that the C3a-receptor is strongly expressed on ischemic endothelium and that exogenous C3a dramatically enhances endothelial cell permeability. Next, we assessed the effect of tPA administration on brain edema and hemorrhage in a transient model of focal cerebral ischemia in C57BL/6 mice. We found that intravenous tPA exacerbates brain edema and hemorrhage in stroke, and that these effects are abrogated by a small-molecule antagonist of the C3a receptor. These findings establish for the first time that intravenous tPA dramatically upregulates complement cascade activation in ischemic brain and that pharmacologic complement inhibition protects against the adverse effects of tPA-mediated thrombolysis in stroke.

## Introduction

The occlusion of a cerebral artery incites a series of deleterious biochemical cascades which disrupt the blood-brain-barrier and engender downstream neuronal, glial, and vascular injury. Among the numerous pathways which contribute to this ischemic brain injury, the complement cascade plays a critical role at the intersection of the immune and inflammatory axes [11]. The cleavage product of the central component of the complement cascade, the C3a anaphylatoxin, promotes endothelial activation, permeability, and inflammatory cell infiltration in stroke [22]. Furthermore, genetic and pharmacologic complement inhibition confers robust and sustained neuroprotection [3, 4]. Complement therefore represents a promising therapeutic target for the treatment of stroke.

In stroke, the central complement component C3 is predominantly cleaved through the mannose binding lectin (MBL) pathway [5, 6]. Beyond the three canonical proximal complement activation pathways, extra-convertase (extrinsic) cleavage of C3 and C5 has also been described [7]. Both thrombin and plasmin, proteases present in the ischemic brain milieu, have been shown to cleave C3 *in vitro* [8]. However, the extent to which such an extrinsic complement activation pathway plays a role in stroke has not been explored. This is particularly relevant because intravenous tissue plasminogen activator (tPA), which cleaves fibrin by generating plasmin from its precursor plasminogen, remains the only approved pharmacologic treatment for stroke patients. Although timely thrombolysis may arrest infarct progression and improve post-stroke outcome, universal use of intravenous thrombolysis is limited by the propensity of tPA to cause vascular injury and brain hemorrhage. The potential neurotoxicity of tPA has been well documented by both in vitro as well as in vivo studies. For instance, Flavin et al. have reported that exogenous tPA exogenous induced apoptosis in cultured neurons. In another report, it was suggested that tPA stimulates apoptosis in ischemic human brain endothelium and in mouse cortical neurons [9]. Additionally, it has been reported that tPA increased neuronal damage following cerebral ischemia in both wild-type and tPA KO mice[10]. tPA-induced neurotoxicity is not restricted to ischemia models, as it has also been demonstrated that tPA deficient mice have shown reduced cortical lesion and attenuated brain edema following traumatic brain injury (TBI) when compared with their wild-type littermates [11]. In addition to the known diverse mechanisms implicated in the adverse effects of tPA on ischemic brain [9, 10, 12], tPA also likely exacerbates deleterious complement activation. Complement inhibition therefore represents an ideal strategy to limit the secondary injury associated with tPA.

In the present study, we first established the mechanism by which tPA promotes C3 cleavage and investigated the effect of C3a on endothelial cell permeability *in vitro*. We then evaluated whether this extrinsic complement activation pathway is functionally-relevant in stroke by demonstrating that tPA promotes brain edema and hemorrhage in our stroke model and that complement inhibition protects against these adverse effects of tPA in stroke. This study provides the first evidence for a critical inter-connection between the complement and coagulation pathways in stroke which may be pharmacologically modulated to minimize the deleterious effects of intravenous tPA.

## Materials and methods

### Ethics statement

Experiments were performed in accordance with the guidelines for the care and use of laboratory animals and approved by the Institutional Animal Care and Use Committee of the University of Pittsburgh. Surgical procedures were performed under isoflurane anesthesia, and all efforts were made to minimize suffering.

### Animals and reagents

Male C57BL/6 mice and MBL-null (B6.120S4-*Mbl1*^*tm1Kata*^*Mbl2*^*tm1Kata*^/J) mice (25-28g; Jackson Laboratories, Bar Harbor, Maine) were randomly allocated between treatment groups. Animals were allowed free access to food and water in a temperature-controlled environment (25^°^C) before and after surgery. Pregnant (day 15) Swiss Webster mice (Jackson Laboratories) were the source of primary neuronal cells.

C3a receptor antagonist SB290157 (C3aRA) and human C3 were obtained from EMD Millipore. Human Lys-plasmin and Glu-plasminogen were purchased from Innovative Research, and tissue plasminogen activator (tPA; Activase) was from Henry Schein. Human/mouse plasma and FITC-dextran (40kDa) were produced by Sigma-Aldrich.

### Middle cerebral artery occlusion

Focal cerebral ischemia was induced in mice by transient middle cerebral artery occlusion (MCAO) as previously described [13]. Anesthesia was induced with isoflurane in 70% N_2_O and 30% O_2_ titrated to 1.5% during surgery. Rectal temperature was maintained between 37.0–37.5^°^C with a temperature-controlled heating pad. A silicon-coated, 6–0 nylon suture was introduced into the ECA and advanced up the ICA to occlude the middle cerebral artery (MCA). Following 60 minutes, the filament was removed to restore perfusion. Cerebral blood flow was measured using laser Doppler flowmetry as previously described [14]. tPA (10mg/kg) or volume-equivalent sterile water was administered intravenously (i.v.) at the time of reperfusion, and C3aRA (1mg/kg in 1.16% DMSO/PBS) or vehicle (1.16% DMSO/PBS) was injected intraperitoneally 45 minutes prior to ischemia. All injections were allocated randomly and performed in a blinded fashion. Sample size was based on our historic data and variance in prior studies using this model[15]. This analysis found that that 12 animals per arm would be required to detect a 20% difference in neurological score between groups with α<0.05, and β = 80%. Given an expected mortality rate of approximately 20%, 15 animals per arm were used.

### tPA-mediated C3 cleavage

Human C3a concentration was quantified using commercially-available ELISA (BD Biosciences). A mouse C3a ELISA was developed by our laboratory using commercial antibodies (BD Biosciences). Briefly, rat anti-mouse C3a capture antibody was coated in a 96-well plate overnight at 4^°^C. Following incubation, the plate was washed and blocked with 5% skim milk. The samples were added to the well and C3a was detected by biotin rat anti-mouse C3a antibody. The plate was read at 450nm (Synergy H1, BioTek) after TMB substrate and stop solution were added.

C3 cleavage experiments were performed by incubating native human C3 (0.02μg/ml) in PBS in the absence or presence of plasmin (0.1–10μg/ml) at 37^°^C for 90 minutes. C3a generation was assessed by ELISA. To assess the mechanism by which tPA cleaves C3 in plasma *ex vivo*, we incubated tPA (100μg/ml) with human plasma (1:1000, Sigma-Aldrich) with and without α-2 anti-plasmin (Abcam; 100nM) or recombinant CD35 (R&D systems; 20nM) for 90 minutes at 37^°^C. C3a concentration was determined using ELISA.

For assessment of C3a generation *in vivo* in plasma, tPA (10 mg/kg) was injected (i.v.) into C57BL/6 mice. Venous blood was collected into citrate-coated tubes 10 minutes after tPA administration. Plasma C3a levels were measured by ELISA. To quantify C3a generation in ischemic brain, tPA (10 mg/kg) was injected (i.v.) at reperfusion. After 24 hours, mice were anesthetized and brains were perfused with PBS, harvested and homogenized. C3a generation was quantified using ELISA.

### Cell culture and oxygen-glucose deprivation (OGD)

Culture of primary cortical neurons (PCNs) was performed as previously described [16]. Briefly, cerebral cortex of Swiss Webster mouse embryos (E15) were freed from meninges and separated from olfactory bulb and hippocampus. Trypsinized cells were suspended in neurobasal medium with 2% (vol/vol) B27 supplement, 0.5mM glutamine, 100 units/mL penicillin and streptomycin (GIBCO), and seeded at a 3x10^5^/500μl in 24-well plates. Experiments were performed on day 7 of culture. For oxygen glucose deprivation (OGD), the culture medium was replaced by glucose-free Earle’s balanced salt solution (EBSS), and cells were placed in a temperature-controlled anaerobic chamber containing a gas mixture composed of 5% CO_2_, 10% H_2_ and 85% N_2._ Control cells were incubated in EBSS with glucose in a normoxic incubator for the same period. After 3 hours, OGD was terminated by a return to normal culture conditions for 24 hours.

The immortalized mouse brain endothelial cell line (bEnd.3) was obtained from ATCC (Manassas, VA) and cultured in DMEM medium with 10% FBS, 2mM glutamine and 1x nonessential amino acid. The cells were incubated in a CO_2_ incubator with 5% CO_2_ at 37^°^C. To model *in vitro* ischemia, we cultured cells in OGD medium for 1 hour in an anaerobic chamber. Following OGD, the cells were returned to a normoxic incubator under 5% CO_2_/95% air for 1, 2 or 24 hours with complete medium. Normoxic bEnd.3 cells served as controls.

### C3aR RNA and protein expression studies

For real time polymerase chain reaction, total RNA was isolated from PCN, bEnd.3 cells, and mouse brain tissue by TRIzol reagent. RNA was transcribed to cDNA using iScript Reverse Transcription Supermix (Bio-Rad), and real-time PCR was performed according to the SsoAdvanced Universal Probes Supermix reagents kit protocol supplied by the manufacturer (Bio-Rad). Mouse C3ar1 primers and probe were obtained from Applied Biosystems.

For Western blot (WB), total protein from bEND.3 cells and mouse brain homogenates were run on NuPAGE 4–12% Bis-Tris gel (Invitrogen) and transferred to nitrocellulose membranes (Bio-Rad). The membrane was probed with anti-C3aR antibody (Santa Cruz Biotechnology, 1:200). Following incubation with the secondary antibody from Li-Cor Biosciences, fluorescent signals were detected by an Odyssey scanner (Li-Cor).

### C3aR immunocytochemistry

bEnd.3 cells (3x10^5^/well) were plated in 24-well glass plate (In-Vitro Scientific) and placed in a CO_2_ incubator overnight then fixed using 4% PFA for 20 minutes at room temperature. Endothelial cells were subjected to OGD as described above and fixed immediately. Samples were blocked with 10% horse serum in TBS with 0.5% Triton X-100 for 1 hour. After washing the cells with TBS, primary anti-mouse C3aR antibody (Santa Cruz Biotechnology, 1:50) diluted in 2% horse serum was added \and incubated overnight at 4^°^C. Next, cells were incubated for 2h with fluorescein anti-mouse secondary antibody (Vector, 1:200), mounted in mounting media with DAPI (Vector), and analyzed using fluorescence microscopy (IX81, Olympus).

### Cell-death assay

Cell death was assessed by LDH assay according to the manufacturer’s instructions (Roche Products). Serial concentrations of murine C3a (R&D Systems, 0-100nM) were incubated with PCN and bEnd.3 cells subjected to OGD followed by 24h re-oxygenation. The reaction mixture (100 μl) was added to conditioned media (100μl) removed from culture wells. Sample absorbance was measured (Synergy H1, BioTek) at 490nm after 15 minutes of room temperature incubation. The same volume of blank medium was used as background control.

### Endothelial cell permeability assay

To assess the effect of C3a on endothelial cell permeability following OGD, bEnd.3 cells were grown on 0.4μm pore membrane cell culture inserts (Nunc) on 24-well plates. Cells (2x10^5^/insert/mL) were cultured in DMEM medium with 10% FBS on upper side of the insert. After the cells reached confluence, 1 mg/ml FITC-dextran (40kDa, Sigma-Aldrich) was added to the upper chamber containing 100nM of C3a in 100 μl of EBSS. EBSS (300μl) was added to the lower chamber to prevent the formation of an oncotic pressure gradient. The cells in EBSS were exposed to OGD for 1 hour, followed by re-oxygenation for 1h in normal CO_2_ incubator. Medium was then collected from the lower chamber and the amount of FITC-dextran was measured using a BioTek fluorimeter at excitation and emission wavelengths of 492nm and 520nm.

### Infarct volume and neurological assessment

Following 24 hours of reperfusion, animals were anesthetized and decapitated. Brains were removed and chilled on ice. Coronal sections (1-mm-thick) were cut using a mouse brain matrix (Roboz Surgical Instrument Co), and stained with 2% TTC (Sigma Co, St Louis, Missouri) at 37^°^C for 20 minutes. Infarct volume was calculated by integrating the infarct area over 6 coronal sections using ImageJ. To control for edema, infarct volumes were expressed as a percentage of the contralateral hemisphere [17]. Absolute edema was calculated as the difference in area between ischemic and non-ischemic hemispheres calculated over all coronal sections [18]. Relative edema was reported as the quotient of absolute edema over absolute infarct volume.

Neurological deficits were assessed as previously described by a blinded observer, using the following scoring system [19]: 0, no observable deficits; 1, forelimb flexion; 2, forelimb flexion and reduced resistance to lateral push; 3, forelimb flexion, reduced resistance to lateral push, and unilateral circling; 4, forelimb flexion and ambulation inability/difficulty.

### Hemoglobin assay

A spectrophotometric hemoglobin assay was employed to quantify the extent of intracerebral hemorrhage[20]. In brief, mouse brains were homogenized, sonicated, and centrifuged, and methemoglobin in the supernatants was converted (using Drabkin's reagent) to cyanomethemoglobin. Concentration was assessed by measuring optical density at 540nm (Synergy H1, BioTek). Bovine erythrocyte hemoglobin (Sigma) was used as a standard.

### Statistical analyses

Quantitative data are presented as mean ± SEM. Difference among multiple groups was analyzed by one-way ANOVA followed by Tukey’s multiple comparison test. Comparison between two groups was based on Student’s *t* test. A value of *P*<0.05 was considered significant.

## Results

### Plasmin cleaves C3 in a dose-dependent manner

**Fig 1:**
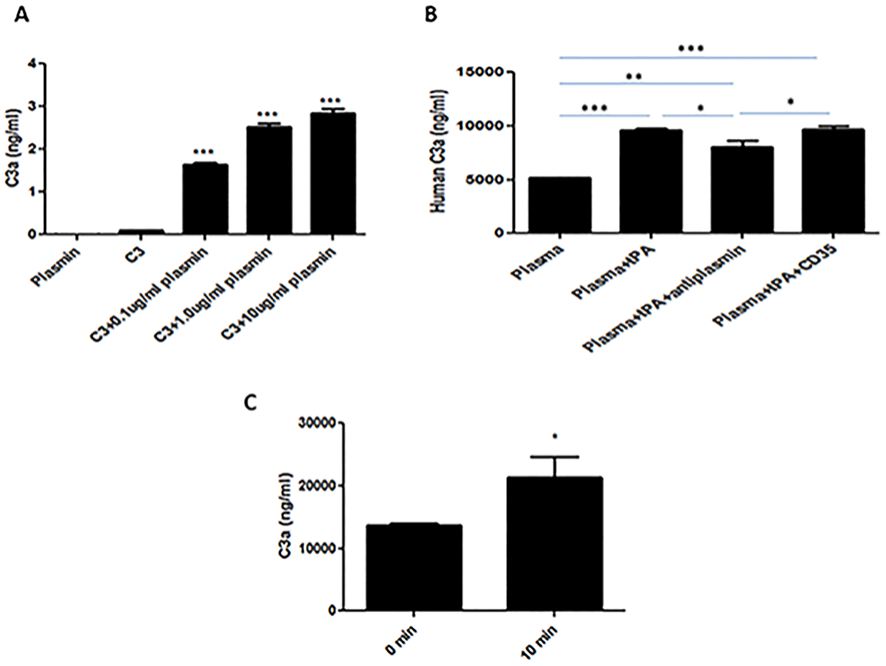
tPA promotes C3 cleavage through a plasmin-mediated extrinsic pathway. **(A)** C3 protein was incubated with plasmin. Plasmin-mediated C3a production increased in a dose-dependent manner. ***P<0.001 vs. control (n = 3). **(B)** tPA promotes C3 cleavage in human plasma, and this process is suppressed by α2-antiplasmin but not by CD35. *P<0.05, **P<0.01, ***P<0.001 vs. controls (n = 3). **(C)** Following intravenous tPA infusion in C57BL/6 mice in the absence of ischemia, a significant increase in plasma C3a levels was observed. *P<0.05 (n = 3).

To demonstrate the extent to which plasmin cleaves C3, serial concentrations of plasmin were incubated with human C3. C3a was generated in a dose-dependent manner (Fig 1A), with no significant C3a detected in the presence of plasmin or C3 alone.

### tPA promotes C3 cleavage in plasma through a plasmin-mediated mechanism

We then set out to demonstrate that tPA mediates plasmin-dependent cleavage of C3, independent of the C3 convertase. tPA (100μg/mL) was incubated with human plasma in the presence or absence of α2-antiplasmin (100nM) or recombinant CD35 (20nM), an inhibitor of complement activation. We found that the addition of tPA to plasma generates C3a (Fig 1B), and that this process is suppressed by α2-antiplasmin but *not* by CD35. As CD35 (Complement receptor-1) inhibits complement activation by binding C3b to prevent the assembly of the C3-convertase [21], these data suggest that tPA promotes C3 cleavage through a plasmin-mediated pathway, independent of the C3-convertase.

We next evaluated whether tPA-mediates C3 cleavage in murine plasma by injecting non-ischemic C57BL/6 mice with intravenous tPA and sampling venous blood after 10 minutes. We noted a significant increase in plasma C3a concentration following tPA injection (Fig 1C). Therefore, tPA promotes plasma C3 cleavage both *ex vivo* and *in vivo*, and the co-factor necessary for plasminogen conversion is present in plasma.

### C3aR is strongly expressed in ischemic brain tissue and endothelial cells

**Fig 2:**
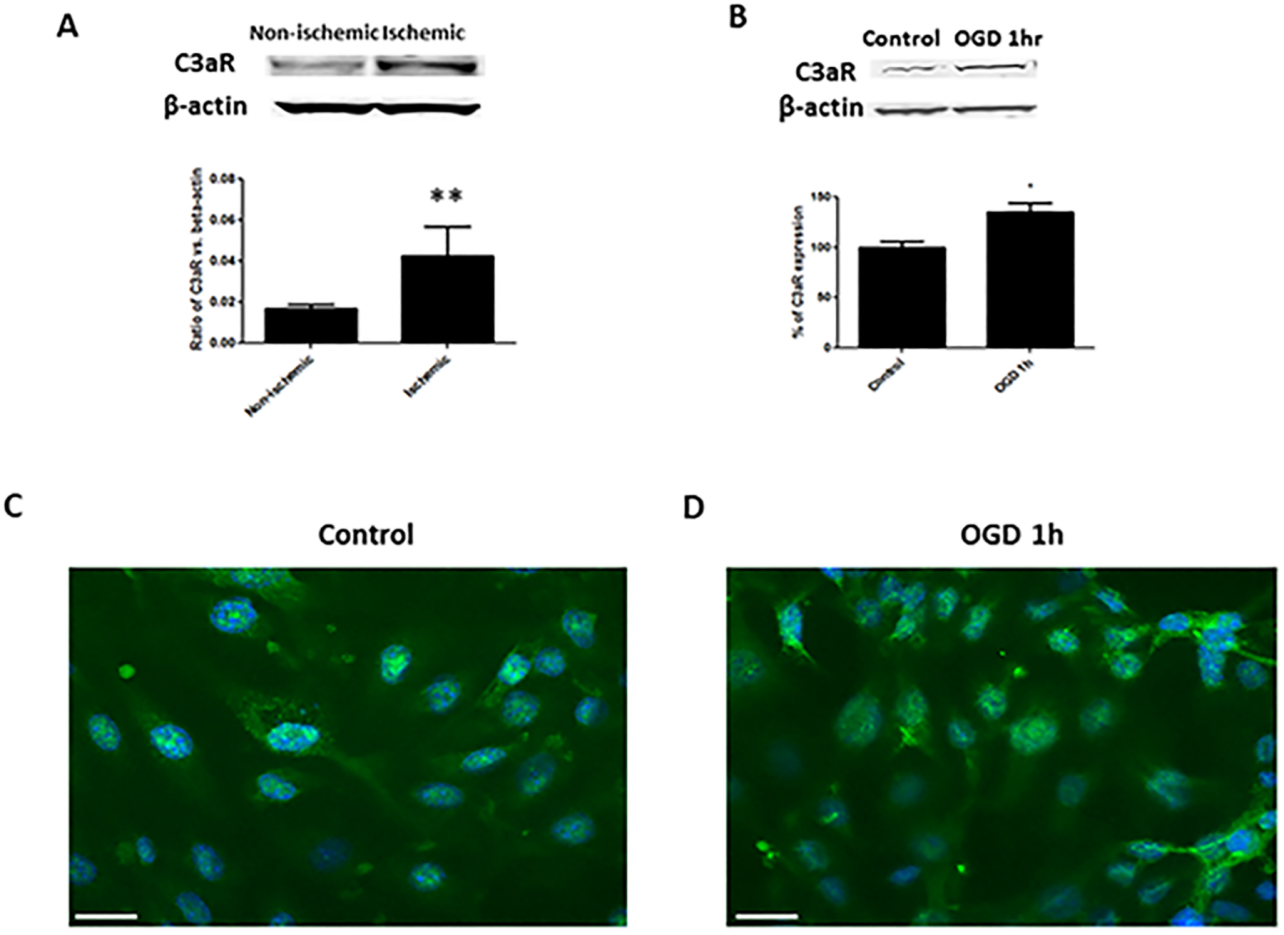
C3aR is strongly expressed in ischemic brain tissue and endothelial cells subjected to OGD. **(A)** WB for C3aR on brain homogenates obtained from mice subjected to MCAO. C3aR protein is strongly upregulated in the ischemic hemisphere. **P<0.01 (n = 5). **(B)** Western blot for C3aR performed on lysates of bEnd.3 cells subjected to OGD demonstrates significantly increased C3aR protein expression relative to non-ischemic controls. *P<0.05 (n = 3). **(C)** Immunocytochemical staining (40X) demonstrates baseline C3aR expression on non-ischemic bEnd.3 cells. **(D)** Following OGD, enhanced C3aR expression (green) is noted. Scale bars = 20 μm.

We next set out to determine the localization of C3aR expression. To investigate whether C3aR is expressed by ischemic neurons, we performed real-time PCR and WB on PCN cultures subjected to OGD as well as normoxic controls. We found that C3aR mRNA and protein were minimally expressed in PCN cultures in both cases. Next, WB performed on ischemic mouse brain lysates demonstrates C3aR protein upregulation in the ischemic hemisphere (Fig 2A).

We next performed WB for C3aR on bEnd.3 cells subjected to OGD. We found strong C3aR expression on ischemic endothelial cells relative to normoxic controls (Fig 2B). Subsequent immunocytochemistry also suggested enhanced C3aR protein expression on bEnd.3 cells subjected to OGD (Fig 2C and 2D).

### C3a does not directly induce neuronal or endothelial cell death but promotes endothelial cell permeability

**Fig 3:**
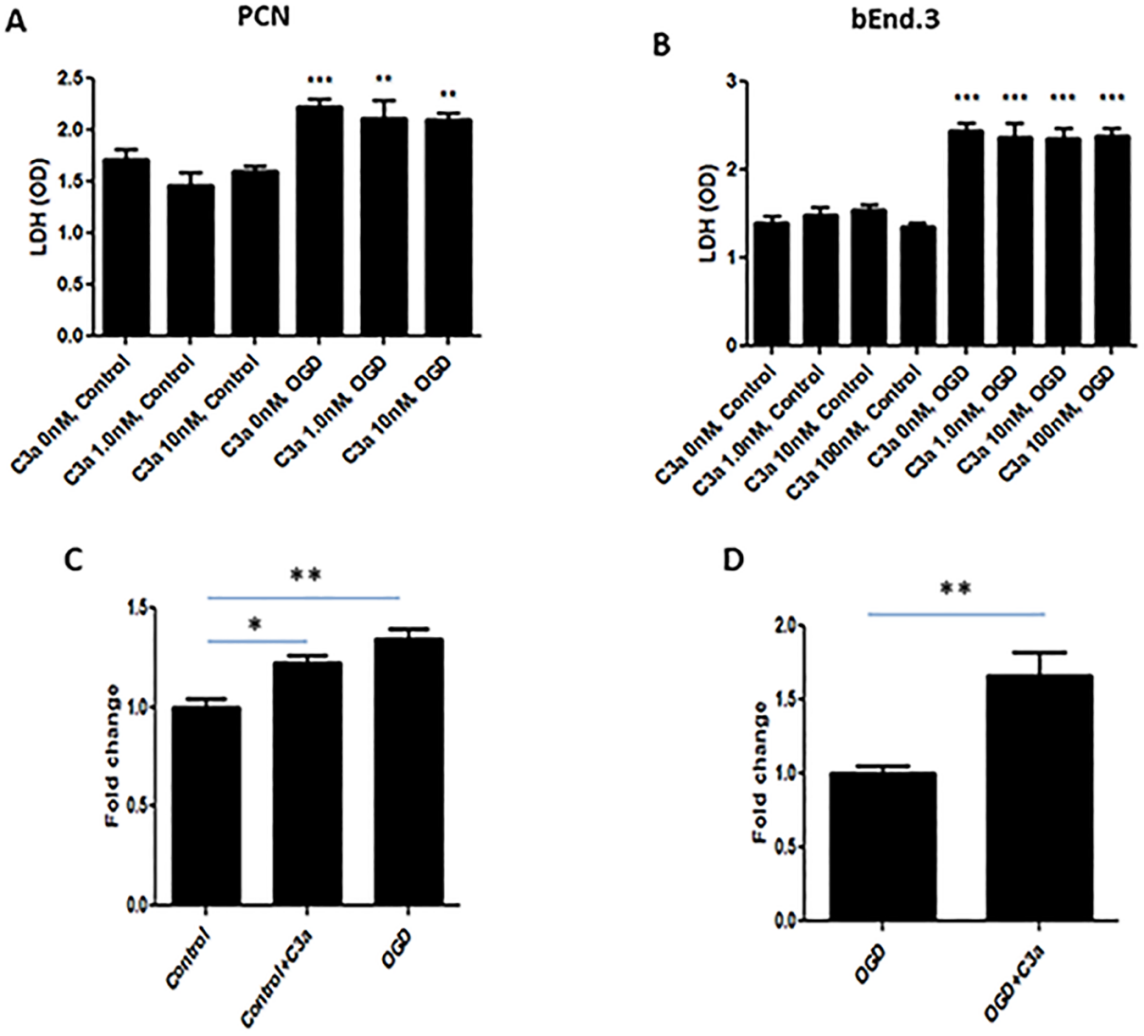
C3a does not directly cause neuronal or endothelial cell death but modulates endothelial cell permeability *in vitro*. Recombinant mouse C3a was incubated with PCN and bEnd.3 cells ± OGD. OGD increases LDH release by PCN **(A)** and bEnd.3 cells **(B)**. However, C3a did not affect LDH production by either cell type. **P< 0.01, ***P< 0.001 vs. controls (n = 4–6). We next assessed the effect of C3a on permeability of a monolayer of bEnd.3 cells subjected to OGD. **(C)** In this model, C3a alone as well as OGD increased permeability compared to control non-ischemic cells. **(D)** C3a also increased monolayer permeability relative to OGD alone. *P< 0.05, **P< 0.01 vs. controls (n = 4).

To determine whether C3a induces neuronal and endothelial cell death, mouse C3a protein was incubated with PCN and bEnd.3 cells subjected to OGD as well as non-hypoxic controls. As expected, we found that OGD alone significantly increased LDH release by both cell-types (Fig 3A and 3B). However, administration of exogenous C3a had no effect on LDH production by either neurons or endothelial cells. These results suggest that C3a does not directly influence cell death, and that the adverse effects of C3a occur through a different mechanism.

We then assessed the effect of C3a on cell permeability using a monolayer of endothelial (bEnd.3) cells subjected to OGD. In this model, both C3a and OGD increased permeability of this cell monolayer to FITC dextran compared to control (Fig 3C). Following OGD and reoxygenation, C3a administration exacerbated permeability even further (Fig 3D). Together with the increased expression of C3aR in endothelial cells subjected to OGD, these data suggest that the C3a-C3aR axis plays a crucial role in modulating endothelial permeability both at baseline and after OGD.

### tPA promotes C3 cleavage in ischemic brain through a mechanism independent of MBL

**Fig 4:**
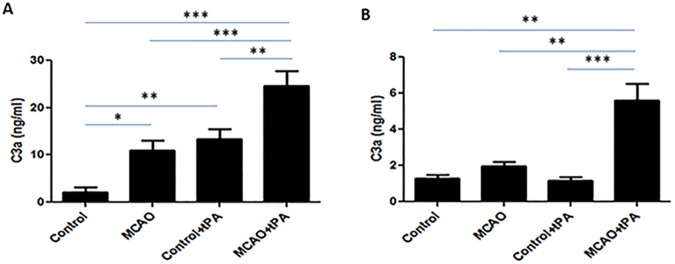
tPA promotes C3 cleavage in ischemic mouse brain through an MBL-independent pathway. Brain homogenates were obtained from mice subjected to MCAO who received i.v. tPA at reperfusion. **(A)** In wild-type mice, a significant increase in C3a concentration was observed in the ischemic hemisphere, with further increases in C3a after tPA administration. **(B)** MBL-null mice did not demonstrate significant C3 cleavage in the ischemic hemisphere; however, tPA does promote a dramatic increase in brain C3a in these mice. *P<0.05, **P<0.01, ***P<0.001 vs. controls (n = 7).

To evaluate the extent to which intravenous tPA promotes C3 cleavage in stroke, brain tissue homogenates were obtained from mice 24 hours following MCAO. As expected, elevated concentrations of C3a was noted in the ischemic hemisphere, with significantly increased C3 cleavage observed following intravenous tPA administration (Fig 4A). In the brains of MBL-null mice, however, ischemia did not enhance C3 cleavage, suggesting that C3 cleavage in ischemic brain occurs through the MBL pathway. In these same MBL-null mice, tPA administration significantly increased C3a concentration in the ischemic hemisphere (Fig 4B). Taken together, these data suggest that tPA promotes direct complement cleavage in ischemic brain through a mechanism independent of the known pathway of ischemia-induced complement activation.

### Pharmacologic inhibition of the C3a-receptor ameliorates post-ischemic tPA-mediated brain edema and hemorrhage

**Fig 5:**
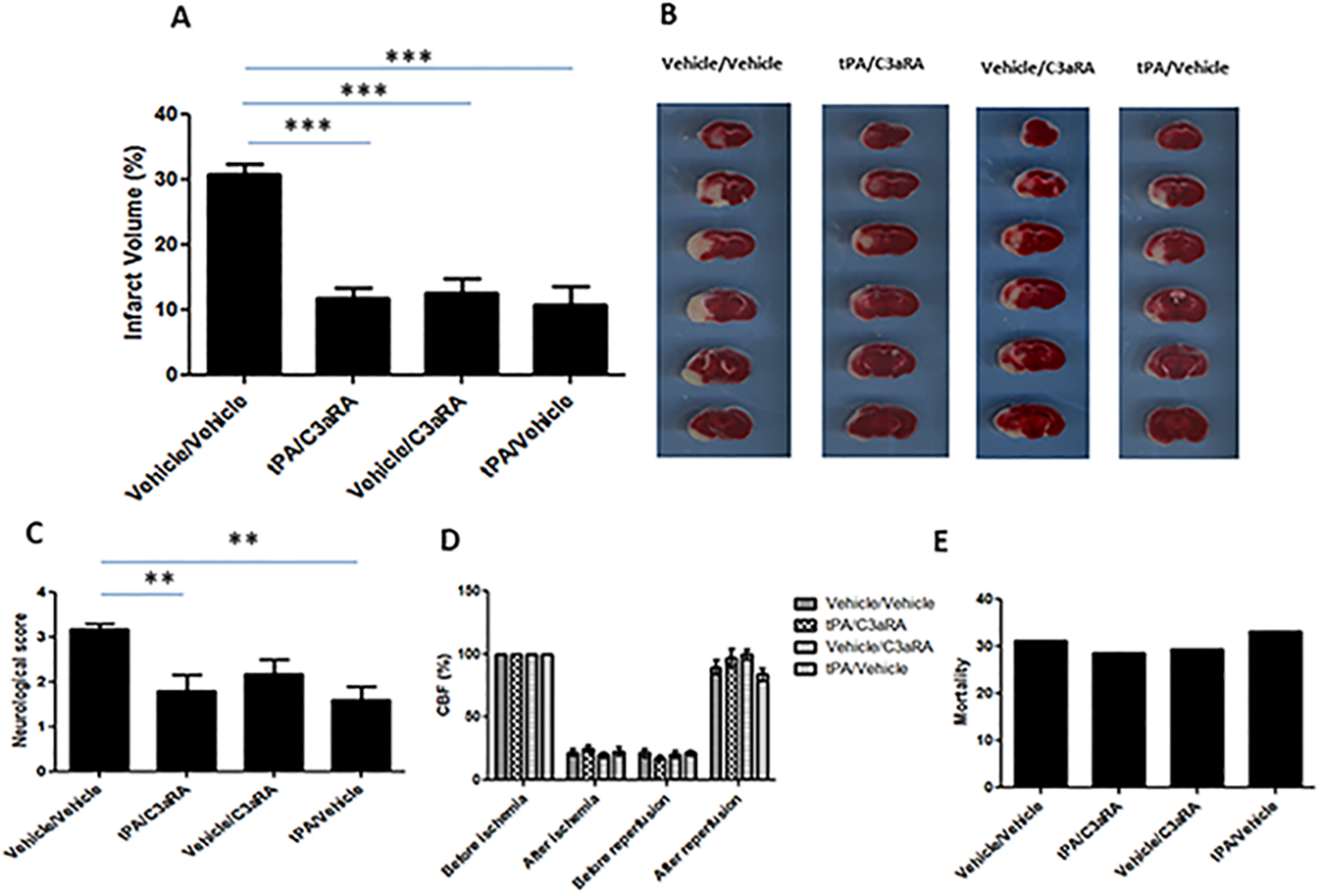
Both tPA and C3aRA confer robust neuroprotection following transient MCAO. **(A)** Following transient MCAO, C3aRA, tPA and the combination of C3aRA/tPA significantly reduced infarct volume relative to vehicle-treated controls. **(B)** Representative TTC-stained coronal sections of brains from mice in each cohort. **(C)** tPA alone or tPA/C3aRA improves neurological function relative to vehicle-treated controls. **(D)** No significant differences in cerebral blood flow were noted among treatment groups. (E) No significant differences in mortality were noted among treatment groups. **P< 0.01, ***P<0.001 vs. controls (n = 10).

We next sought to determine the functional effects of C3aR inhibition in mice subjected to MCAO and treated with tPA. We found that administration of C3aRA, tPA, and the two in combination significantly reduced infarct volume compared to vehicle-treated controls (P<0.001) (Fig 5A and 5B). Additionally, tPA alone or tPA administered in conjunction with C3aRA also enhanced neurological function (P<0.01) (Fig 5C). Importantly, there were no significant differences in cerebral blood flow (CBF) among the treatment groups at any time-point examined (Fig 5D), and mortality did not differ significantly between cohorts.

**Fig 6:**
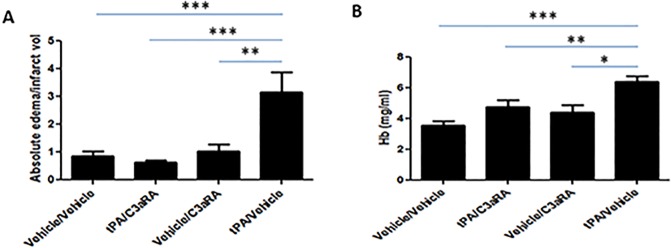
C3aRA ameliorates tPA-mediated brain edema and hemorrhage following MCAO. **(A)** tPA administration significantly increases relative cerebral edema, and this increase was ameliorated by C3aRA. **(B)** tPA significantly increased cerebral hemorrhage following MCAO, an effect that was suppressed by antagonism of the C3a receptor. *P<0.001, **P<0.01, ***P<0.001 vs. controls (n = 10).

We then assessed whether C3aR inhibition would protect against tPA-mediated brain edema and hemorrhage in our model. We found that post-ischemic tPA administration increased relative brain edema in the ipsilateral hemisphere, and that this edema was suppressed by C3aR antagonism (P<0.001) (Fig 6A). Furthermore, tPA significantly increased brain hemorrhage, and C3aRA treatment greatly reduced the extent of hemorrhage (P<0.01) (Fig 6B). Taken together, these data suggest that pharmacologic inhibition of the C3aR effectively protects against these deleterious side-effects of intravenous tPA.

## Discussion

**Fig 7:**
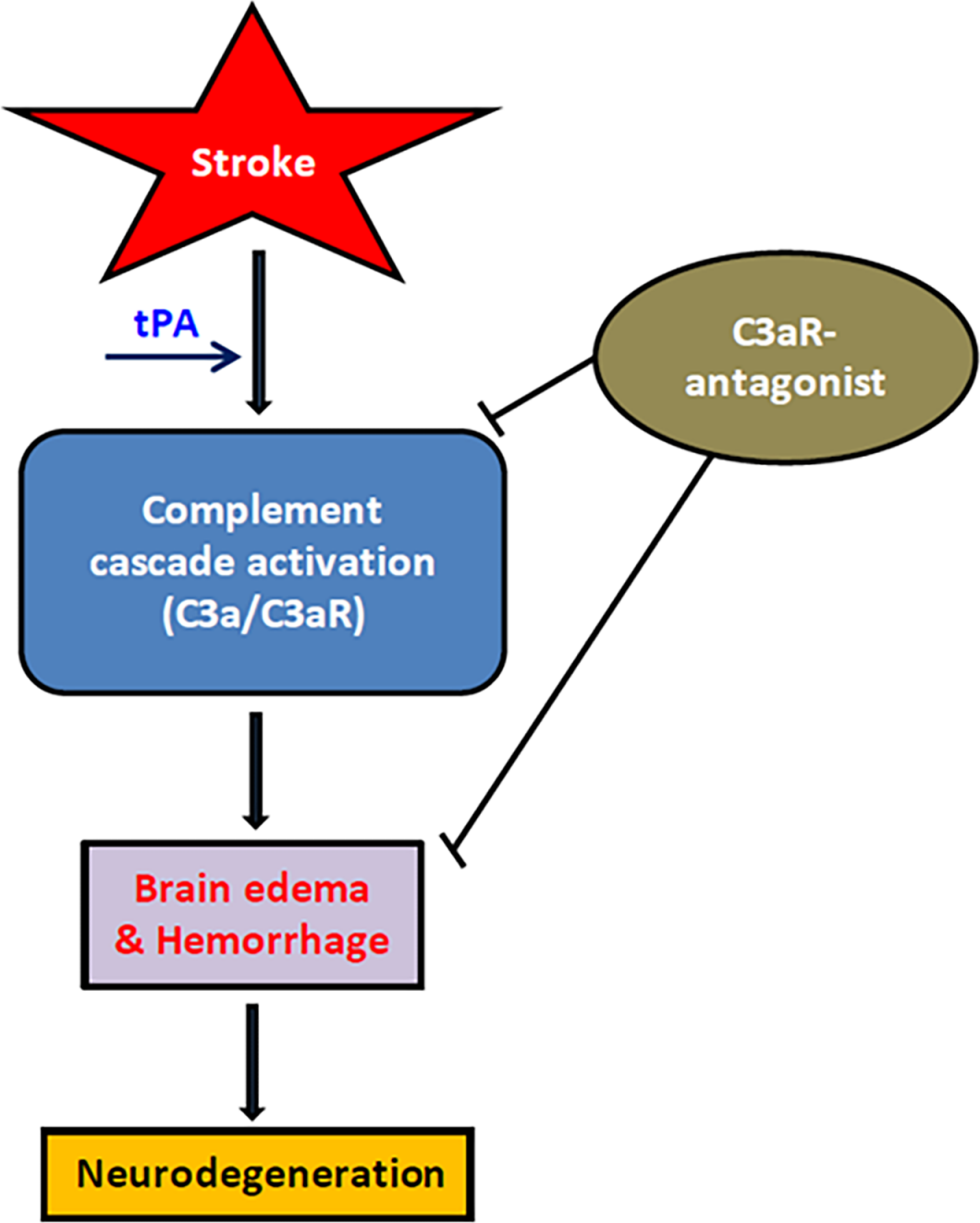
tPA-mediated complement cascade activation in stroke. Schematic depicting the interplay between cerebral ischemia and tPA, resulting in complement C3a generation, and subsequent brain injury.

The present study demonstrates that intravenous tPA triggers complement C3 cleavage in ischemic brain through an extrinsic pathway. Additionally, we show that tPA exacerbates cerebral edema and hemorrhage in our stroke model, analogous to findings in human stroke patients. Finally, we demonstrate that pharmacologic antagonism of the C3a receptor ameliorates the adverse effects of tPA and represents a promising potential therapeutic strategy for stroke (Fig 7).

Although tPA remains the only approved pharmacologic treatment for stroke, its application is limited to patients who present within a limited time-window. This is due in part to the propensity of tPA to promoted hemorrhage and brain edema, which occur more frequently when tPA administration is delayed [22, 23]. However, recent clinical trials of endovascular thrombectomy have shown that even delayed reperfusion may arrest stroke evolution and dramatically improve outcome in select patients, arguing that salvageable penumbral brain tissue often persists well beyond the accepted strict therapeutic window for i.v. tPA [24–27]. Therefore, strategies to mitigate the deleterious effects of tPA while preserving its beneficial thrombolytic effect might potentially allow for safe administration of this effective thrombolytic to a larger subset of stroke patients.

Several studies have probed the mechanisms underlying the adverse effects of tPA in stroke. First, a seminal study demonstrated that tPA-deficient mice exhibit smaller infarcts than wild-type controls [10], suggesting for the first time that tPA may actually promote ischemic brain injury. Several mechanisms including damage to the integrity of the BBB as well as direct neuronal toxicity have been implicated [10, 28]. One major theme of subsequent investigation focused on the ability of tPA to upregulate the expression of matrix metalloproteinases (MMPs), which degrade the structural proteins of the BBB, engendering edema and predisposing to hemorrhage [29]. In a parallel line of work, Yepes *et al*. showed that endogenous tPA activity in the perivascular tissue following cerebral ischemia induces opening of the BBB through a receptor-mediated mechanism [28]. This group also showed that tPA administered into the lateral ventricle causes BBB-breakdown through a PDGF-α receptor-mediated mechanism [30]. Although these studies delineate an important mechanism of tPA-mediated BBB dysfunction, they focus on the effects of endogenous tPA as well as the administration of exogenous tPA into the ventricle and ignore the critical interaction of tPA with the cerebral vasculature. The overall evidence suggests that the deleterious effects of tPA in stroke occur through pleiotropic mechanisms.

Complement has been well-established as a critical mediator of brain injury in stroke [1, 13]. In addition to the three canonical complement activation pathways, a number of extrinsic pathways have recently been described. The best-understood of these pathways involves thrombin, which cleaves C5 directly even in the absence of C3 [7]. Plasmin, another protease present in the ischemic brain milieu, has also been shown to cleave C3 and C5 *in vitro* [8]. Little attention has been focused on whether this particular complement activation pathway occurs *in vivo*. A single prior study demonstrated an increase in plasma complement activation following tPA administration in patients undergoing coronary thrombolysis [31]. However, no prior study has examined whether tPA-mediated complement activation occurs in ischemic brain.

The present study establishes for the first time that tPA administration dramatically enhances C3 cleavage in ischemic brain tissue. Even in MBL-null mice, in whom ischemia-induced complement activation is abrogated [15], tPA administration results in the generation of significant C3a in ischemic brain. This suggests that tPA-mediated complement cleavage occurs independently of MBL, the primary proximal complement activation pathway in stroke. Although we have not administered C3aRA to MBL-null mice subjected to cerebral ischemia, given the lack of significant post-ischemic complement activation observed in brains of these mice, we would not expect C3a receptor inhibition to be protective. Additionally, in the setting of tPA administration, given the significant increase in cerebral C3a, we would expect C3aRA administration to be protective and these experiments are planned for the future. Interestingly, in WT mice, administration of tPA promotes C3 cleavage even in the absence of ischemia. This suggests that intravascular tPA may influence the permeability of the blood-brain-barrier even in the absence of ischemia, and further work is necessary to delineate the mechanisms associated with this process.

Our *in vitro* findings demonstrate that tPA-mediated C3 cleavage occurs through a plasmin-mediated mechanism, as the addition of α2-antiplasmin, a plasmin inhibitor, suppresses C3a production. The fact that CD35 does not similarly suppress C3 cleavage confirms that the C3 convertase is not involved in tPA-mediated C3 cleavage. Importantly, prior work has demonstrated that plasminogen receptors are upregulated at sites of cellular injury[32], supporting the concept that tPA- mediated conversion of plasminogen to plasmin might be enhanced locally at sites of injury such as at the ischemic cerebral endothelium.

The present study also confirms the neuroprotective effect of complement inhibition in stroke and represents the first effort to evaluate the functional effects of complement inhibition following tPA administration [1, 2, 5, 13, 15, 33]. In mice subjected to MCAO and treated with tPA, C3a generation is markedly increased despite a reduction in infarct volume. A reduction in infarct size following tPA administration has been previously reported in several MCAO models and likely relates to improved post-ischemic reflow [34], although we only monitored CBF through the immediate peri-operative period and therefore cannot confirm this supposition. Nevertheless, the observation of worsened relative brain edema and cerebral hemorrhage in spite of reduced infarct volume recapitulates clinical observations in human stroke patients [35]. We propose that the pathogenic effects of the C3a anaphylatoxin contribute substantially to the adverse effects of tPA in ischemic brain. Although the specific effect of C3a on brain edema has not previously been investigated in stroke, other experimental disease models have been used to convincingly demonstrate that the C3a/C3aR axis modulates inflammatory cell infiltration and endothelial permeability [36]. In addition to an exacerbation of brain edema, increased vascular permeability likely also contributes to the hemorrhage seen following tPA administration. Reperfusion into ischemic and permeable microvessels permits extravasation of red blood cells across the vasculature into adjacent brain tissue [37]. Further work is necessary to delineate the specific mechanisms responsible for the reduction in edema and hemorrhage observed following C3a receptor inhibition.

This study suffers from several important limitations. First, our evaluation of outcome at a single 24 hour time-point represents a substantial limitation. Further work to evaluate the long-term functional effects of complement inhibition following tPA administration is required and is planned for the future. Additionally, as these experiments were primarily designed to probe the mechanism of tPA-mediated complement activation, we did not perform post-ischemic dosing regimens which will ultimately be critical for translation of anti-complement strategies. Additionally, these experiments were performed using sterile water as the vehicle for reconstitution of the alteplase formulation of tPA. As such, administration of the vehicle alone intravenously may have resulted in a hypo-osmolar environment. We do not believe that this influenced the outcome of our edema analysis, which showed significantly increased relative edema in the tPA treated cohort relative to vehicle treated controls. Nevertheless, future experiments will utilize a true vehicle control injection which includes the non-tPA components of the Alteplase formulation to serve as a true vehicle control. Finally, we utilized an intraluminal filament model with tPA administered at the time of reperfusion. Although this model simulates effective thrombolysis as might occur with mechanical thrombectomy, additional work using both a permanent model and an embolic stroke model are critical. In particular, an embolic model of MCAO represents the most clinically-relevant model particularly in the setting of tPA-administration and these experiments are planned for the future.

In summary, the present study is the first to identify the critical interplay between the complement and coagulation cascades that occurs following intravenous tPA administration in stroke. This study demonstrates that tPA promotes C3 cleavage through an extrinsic pathway, and the C3a anaphylatoxin thus generated promotes post-ischemic brain hemorrhage and cerebral edema. Pharmacologic inhibition of the C3aR abrogates these deleterious effects of tPA on the ischemic brain and represents a promising therapy for the treatment of stroke patients.
